# Does Blame Impede Health Recovery After Transport Accidents?

**DOI:** 10.1007/s12207-015-9215-5

**Published:** 2015-02-17

**Authors:** Nieke A. Elbers, Alex Collie, Arno J. Akkermans

**Affiliations:** 1 Faculty of Law, Department of Civil Law, VU University, De Boelelaan 1105, 1081 HV Amsterdam, The Netherlands; 2 Faculty of Psychology and Education, Department of Clinical Psychology, VU University, Amsterdam, The Netherlands; 3 Institute for Safety, Compensation and Recovery Research, Monash University, Level 11, 499 St Kilda Road, Melbourne, 3004 Victoria Australia

**Keywords:** Psychologist, Physiotherapist, Health care, Compensation processes, Blame, Injury

## Abstract

Blame towards the wrongdoer can be a source of distress for people who are injured in a transport accident. The association between blame and psychological stress is well investigated. In contrast, not much is known about blame and health-care utilization. It is important to investigate whether blame is associated with health-care consumption because it may contribute to our knowledge about what factors have an effect on recovery after transport accidents. The current study involved a total of 2940 participants, who were selected from a compensation database in Victoria, Australia. Health-care utilization, in general, and utilization of psychologist and physiotherapist visits, in particular, were defined as the outcome. In contrast to a previous study, it was found that blaming the other was associated with greater health-care utilization, in general, and psychologists and physiotherapist visits, specifically. Another relevant finding was that, although the study involved a sample that was created to show an equal ratio of blame/no-blame, 61 % blamed the other driver; therefore, blame may be a motive to lodge a claim. Finally, we discuss the role that psychologists and claim managers could play in reducing feelings of blame in order to reduce health-care utilization and possibly improve recovery.

## Introduction

The aftermath of being injured in a transport accident can be very stressful: 10–25 % of those injured in motor vehicle crashes suffer from long-lasting psychological complaints (Renssen, 2002). Additionally, those injured may suffer from poorer mental health because they are hampered by feelings of blame towards the person who caused the accident (Sullivan, Davidson, Garfinkel, Siriapaipant, & Scott, 2009). The psychology of blaming the other after a motor vehicle accident has been investigated previously and is consistently found to be associated with poorer mental health and greater distress (Delahanty et al., 1997; Hickling, Blanchard, Buckley, & Taylor, 1999; Ho, Davidson, Van Dyke, & Agar Wilson, 2000; Littleton et al., 2012). In contrast, not much is known about the association between blame and health-care utilization. It is important to investigate this relationship because it can possibly generate knowledge about recovery after a transport accident.

To our knowledge, only one study has investigated the association between blame and health-care utilization. However, this was not the primary aim of that study; the research question was more general about the effect of compensation on health, taking various factors such as blame into account. Patients were asked how many times they had visited particular health-care professionals in the previous 3 months. The relevant health-care professionals were general practitioners, medical specialists (including surgeons and physicians), psychiatrists, physiotherapists, chiropractors, massage therapists and others. The study did not show an association between blame and health-care service use (Harris, Murgatroyd, Cameron, Young, & Solomon, 2009). Given that blame is consistently found to be associated with psychological distress, one could reasonably expect that blame would also result in greater health-care service use. Thus, on the question at issue, there is some disagreement in the literature at present.

An explanation for the absence of significant results in Harris et al. (2009) could be due to certain methodological factors. For example, in their study, health-care utilization was measured over the past 3 months, which may not be long enough to find an effect. Furthermore, the study included both transport and work accidents, which may have confounded the findings; in that work accidents may involve a different perspective of blame because the responsibility of the accident is often less clear than that in transport accidents. Thirdly, health-care utilization was self-assessed by the participants so the outcome might be subjected to recall bias. Finally, the research may not have found an effect because they measured health-care utilization, in general, not specifying different services; blame may only have an effect on specific services, such as psychologist visits.

We sought to address these potential methodological limitations evident in the prior research in the current study. A homogeneous sample was selected consisting of participants injured in transport accidents. Participants were derived from a population-based database, thus minimizing selection bias. The outcome health-care utilization was investigated for a time period of 12 months post injury. Moreover, in terms of outcome, a distinction was made between psychologist and physiotherapist services. It was hypothesized that blame would be positively associated with both health-care service use, in general, and psychologist visits, specifically.

## Method

### Participant Selection

The participants were adults injured in transport accidents, who were selected from the Compensation Research Database (CRD), held at the Institute of Safety Compensation and Recovery Research in the state of Victoria, Australia (Ruseckaite, Gabbe, Vogel, & Collie, 2011). The CRD is a de-identified database of people who were involved in a transport accident and who lodged a compensation claim with the Transport Accident Commission (TAC) in Victoria. In Victoria, there is a no-fault compensation scheme for transport accidents, which means that people are eligible to lodge a claim regardless of fault. They can also lodge a common law claim if they are seriously injured and can demonstrate that they are not at fault. The majority of claimants in the database have only a no-fault claim without proceeding with a common law claim (about 95 %). The CRD holds claims data from 1 January 1987 onwards.

Participants with an accident date between January 2010 and July 2011 were selected. January 2010 was chosen as the lower boundary because blame was not recorded before that. July 2011 was set as the upper limit because data until July 2012 were available, and the follow-up was chosen to be 12 months. Only car drivers in two-vehicle accidents involved in no-fault claims were included in order to create an equal distribution of blame. Subsequently, the following exclusion criteria were applied: claimants who died within 1 year after accident (because the follow-up period was set at 1 year), people who were younger than 18 years old and victims with catastrophic injury, such as severe brain injury and spinal cord injury (because they are offered a more intensive care and support programme compared to people with other injuries). Claimants whose claim was denied were omitted, in that in such cases, the outcome measure would not be registered. Finally, claimants for whom blame was ‘unknown’ were excluded, because this could mean that the claimant either did not know or did not answer this question. Ethics committees of VU University and Monash University approved the use of the database and the analyses.

### Data Selection and Definitions

The selected independent variables were gender, age at accident, injury type and severity of injury. The types of injuries that were recorded in the database were, among others, abrasions, amputations, brain injuries, concussions, dislocations, fractures, internal injuries, soft tissue/whiplash injuries, spinal cord injuries and sprains. All injury types except for spinal cord and brain injuries were included in the analysis, but to simplify the reporting, the type of injury was dichotomized into whiplash versus other. Whiplash was chosen because it was the largest category and because professionals and researchers often consider it to be a difficult/deviant and therefore specifically interesting injury. Severity of injury was defined by length of hospital stay (Harris et al., 2009), being the number of consecutive days spent in the hospital if admitted on the day of accident. Blame was recorded in the database as ‘fault’, which was scored by the TAC officer who had asked the claimants whether the other person/organization was at fault. In this study, the word ‘blame’ is used rather than ‘at-fault’ in order to link with the blame literature, which is more abundant than the number of studies on fault. It is acknowledged that blame and at-fault are not necessarily perfectly compatible concepts (e.g. someone can be at-fault but not be fully blamed). However, in practice, these concepts overlap, especially in the case of a dichotomous outcome, which is the reason for the decision to use blame. If the claimants considered the other to be at-fault, it was labelled ‘other-blame’, if ‘no’, it was further called ‘self-blame’. Health-care utilization was defined as the number of visits to general practitioners, surgeons, psychiatrists, physiotherapists, psychologists, speech therapists, chiropractors, osteopaths, optometrists, podiatrists, occupational therapists, vocational counsellors, neurologists, nurses and acupuncturists (Ruseckaite et al., 2011).

### Statistical Analysis

Descriptive statistics were used to describe the participant characteristics. Chi-square and independent *t* tests were used to analyze the associations between the independent variables and blame. A univariate linear regression analysis was conducted to analyze the association between blame and health-care utilization. A multivariate regression analysis was performed to investigate the association between blame and health-care utilization correcting for age, gender, injury type and severity of injury. The stepwise method was used being the preferred method in case there is no theoretical basis to rely on. Separate analyses were done for health-care utilization, in general, and psychologist and physiotherapist services, in particular. Health-care utilization, in general, included psychology and physiotherapy services.

## Results

### Participant Characteristics

After excluding claimants according to the criteria, the sample consisted of 2940 participants. The sample included less women than men (42.4 vs. 57.6 %), and they were, on average, 43.4 years old. The most frequent injury was whiplash injury (49.2 %), 15.9 % of the participants suffered from contusion, and 11.6 % had fractures. One out of five participants (18.8 %) had been admitted to the hospital directly after the accident. The length of hospital stay for all participants (including those not admitted to the hospital) was, on average, 0.7 days. More than half of the participants blamed the other driver (60.9 %).

Three quarters of the participants received health-care services paid by the compensation agency (72 %); the other 28 % either did not use health-care services, or claimed under alternative funding mechanisms, such as the Australian government universal health-care programme (‘Medicare’), or did not exceed the threshold of $564 Australian dollars required by the TAC scheme before TAC benefit payments begin. Those who claimed health-care services used 16.5 services, on average. Only 3.7 % of the sample went to see a psychologist, and 19.1 % used physiotherapy sessions. The average number of treatment visits per injury is displayed in Table 1.

**Table 1:** The average number (and standard deviation) of health-care utilization visits per injury type

Type of injury	Health-care utilization	Psychologist visits	Physiotherapist visits
Whiplash injury	4.7 (18.4)	0.29 (2.9)	4.32 (12.0)
Contusions/abrasions	4.2 (11.4)	0.09 (0.8)	1.45 (7.3)
Fractures	23.7 (47.0)	0.66 (3.1)	9.57 (23.7)
Internal injuries	25.1 (62.7)	0.31 (2.1)	8.68 (21.4)
Brain injury	53.3 (103.4)	2.22 (8.3)	17.3 (38.3)

### Association Between Independent Variables and Blame

Women and men equally often blamed the other person for the accident (*χ* ^2^ = 1.66, *p* = 0.198). Participants who blamed the other were, on average, 2 years younger than those who considered themselves to be responsible (*t*(2086.73) = 2.70, *p* = 0.007). Participants with whiplash injury more often blamed the other (*χ* ^2^ = 133.7, *p* < 0.001). Those who blamed the other spend fewer days in the hospital (*t*(1805.71) = 4.55, *p* < 0.001). The other-blame group used more health-care utilization, in general (*t*(2779.8) = −4.63, *p* < 0.001); more psychologist services (*t*(2797.1) = −3.57, *p* < 0.001); and more physiotherapist services (*t*(2863.4) = −7.24, *p* < 0.001). Sample characteristics subdivided into self-blame and other-blame are shown in Table 2.

**Table 2:** Sample characteristics divided by self-blame (*n* = 1150) and other-blame (*n* = 1790)

Variable	Self-blame (% or SD)	Other-blame (% or SD)	*p* value (*χ* ^2^ or *t* test)
Gender			
Male	505 (40.5 %)	743 (59.5 %)	0.198
Female	645 (38.1 %)	1047 (61.9 %)	
Age (years)	42.7 (20.0)	44.6 (16.2)	0.007
Type of injury			
Whiplash	413 (28.5 %)	1034 (71.5 %)	<0.001
Contusions/abrasions	240 (51.5 %)	226 (48.5 %)	
Fractures	170 (49.6 %)	173 (50.4 %)	
Internal injuries	83 (41.3 %)	118 (58.7 %)	
Brain injury	49 (53.8 %)	42 (46.2 %)	
Hospital admission			
No	882 (36.9 %)	1506 (63.1 %)	<0.001
Yes	268 (48.6 %)	284 (51.4 %)	
No. of hospital days^a^	1.0 (3.4)	0.5 (2.3)	<0.001
Health-care services	9.0 (29.8)	13.6 (36.6)	<0.001
Psychologist	0.2 (1.5)	0.5 (3.0)	<0.001
Physiotherapist	6.4 (18.3)	2.6 (9.9)	<0.001

^a^Severity of injury

### Association Between Blame and Health-care Service Utilization

The univariate regression analyses showed that blame was associated with increased health-care utilization (*β* = 0.07, *p* < 0.001), psychology services (*β* = 0.06, *p* = 0.002) and physiotherapy services (*β* = 0.12, *p* < 0.001, see Table 3). The multivariate regression analyses adjusting for gender, age, type of injury and severity of injury showed that blame was significantly associated with an increased number of health-care services (*β* = 0.11, *p* < 0.001), psychology services (*β* = 0.07, *p* < 0.001) and physiotherapy services (*β* = 0.14, *p* < 0.001, see Table 3).

**Table 3:** Univariate and multivariate regression analyses of blame and health-care utilization

Outcome	*B*	SE	*β*	*p*	*R* ^2^
Univariate					
Health-care services (all)	4.63	1.29	0.07	<0.001	0.004
Psychologist services	0.30	0.10	0.06	0.002	0.003
Physiotherapist services	3.76	0.59	0.12	<0.001	0.013
Multivariate					
Health-care services (all)	7.91	1.15	0.11	<0.001	0.250
Psychologist services	0.36	0.10	0.07	<0.001	0.011
Physiotherapist services	4.55	0.59	0.14	<0.001	0.059

## Discussion

This study investigated whether blame was associated with health-care service use. In accordance with the hypothesis, there was a significant positive association between blame and health-care usage, in general, and with psychologist and physiotherapist services use, specifically. In other words, people who blame another person use more psychologist and physiotherapist services. It could be that blame is part of a mechanism in which recovery may be hampered. It should be noted that the relationship was not very strong because the betas of the regression analyses were around 0.10, which is considered small. This implies that the hamper of recovery, when it occurs, is a multifactorial process.

The current study found a significant association, which does not correspond with the result of the single previous study examining this issue (Harris et al., 2009). However, it was argued that the other study had some limitations, such as a too short post-event period to measure health-care utilization, which may have impeded finding positive results. The current study avoided these methodological issues, perhaps facilitating why a (small) effect was found. However, more research is needed on the question of blame and outcome after transport accidents.

There is another finding that was not part of the hypothesis but is worthwhile discussing. Although the study involved a sample that was created to show an equal ratio of blame/no-blame, 61 % blamed the other driver. An explanation for this relationship could be that blame is a motive to pursue a compensation claim. This is supported by Harris and colleagues (2008, p. 970) and by a qualitative study showing that seeking acknowledgement for the harm that has been inflicted is one of the motives for making a claim (Akkermans, 2009). These two studies mainly concerned fault-based compensation schemes, so it is interesting that blame also seems to be a motivation to claim in no-fault compensation schemes. It could partly explain why some ‘compensation and health’ studies involving a no-fault scheme report percentages between 7 and 25 % of transport victims who were eligible but did not claim (Gabbe et al., 2007; O’Donnell, Creamer, McFarlane, Silove, & Bryant, 2010); that is, they may have blamed themselves, and therefore, it did not feel appropriate to claim. Previous blame studies, such as Delahanty et al. (1997), Hickling et al. (1999) and Ho et al. (2000), did not take claiming into account, which could have affected the findings, because blaming and claiming may be confounders. It seems important to take both concepts into account in future studies.

While aiming to address the limitations of the Harris study (2009), the current study has its own ones, in that the database used did not contain all relevant measures. For example, it would have been interesting to investigate the association between blame and health status by using a health survey, but no such measure was available. Furthermore, the answer scale for at-fault (blame) was dichotomous (yes/no). Although most blame studies use a dichotomous outcome, it would have been more sophisticated if a Likert scale from 1 to 5 was used. The database also did not contain information about pre-injury health characteristics, which is a limitation because health service use *before* the accident is probably associated with utilization *after*. Finally, there was no validated scale for injury severity such as the Injury Severity Score; therefore, it is not certain whether the study correctly adjusted for injury severity by using length of hospital stay.

With respect to the generalizability of the results, it should be stressed that a specific sample was selected consisting of drivers and multiple-vehicle accidents only. This was done in order to create a homogeneous research sample with an equal distribution of blame. Therefore, the results of this study sample cannot be translated to single-vehicle accidents nor to pedestrians, (motor)cyclists and train/tram/car passengers. Nevertheless, ‘car drivers’ in combination with ‘multiple-vehicle accidents’ covered about 30 % of all transport accidents in the database, which is quite a substantial proportion. An additional comment regarding generalizability concerns the fact that the participants in this study were involved in a compensation scheme in which people are compensated regardless of fault. In most countries, transport accidents are compensated in a tort law-based compensation scheme in which people can only lodge a claim if somebody else is liable (at-fault) for the accident. The feelings of blame are assumed to be higher in fault-based schemes so the results may not translate to participants involved in tort law (fault-based) compensation schemes.

To conclude, as blame appears to be associated with increased health-care utilization, which in its turn may be associated with recovery, it could be important to work with survivors about these feelings of blame. For example, providing psychological interventions and teaching victims to cope with these feelings might be useful. Another rather simple solution would be if the wrongdoer offered a sincere apology to the injured person, which has been found to be effective in a review (Hulst, Akkermans, & Van Buschbach, 2014). However, many times, the offender does not have any contact details or does not dare to apologize. In that case, a psychological support organization could mediate the contact between offenders and victims. In the Netherlands, a pilot study was conducted investigating the experiences in such mediation. The contact was either face to face or via letters and was guided by a trained mediator. Both victims and offenders evaluated this service very positively (Hulst et al., 2014). Another venue that could readily facilitate remedial contact between a wrongdoer and an injured person is the compensation agency. A Dutch pilot experiment is currently being set up in which the insurance company sends a letter to the wrongdoer providing the possibility to leave a personal message to the injured person, which would be forwarded to the injured person. Some empathic example phrases are being suggested to guide the writer (Hulst et al., 2014). It could be a simple, inexpensive intervention with potential positive results.

In summary, this study showed that blame was associated with health-care service usage after injury. Although the association was only small and the study had some limitations, blame may be a factor that should be taken into account in future injury research. The injured persons’ recovery might improve if psychologists and claim managers offer support in reducing feelings of blame.

## References

[CR1] Akkermans, A. J. (2009). Reforming personal injury claims settlement: Paying more attention to emotional dimension promotes victims recovery. Available at SSRN: http://ssrn.com/abstract=1333214

[CR2] Delahanty DL, Herberman HB, Craig KJ, Hayward MC, Fullerton CS, Ursano RJ, Baum A (1997). Acute and chronic distress and posttraumatic stress disorder as a function of responsibility for serious motor vehicle accidents. Journal of Consulting and Clinical Psychology.

[CR3] Gabbe BJ, Cameron PA, Williamson OD, Edwards ER, Graves SE, Richardson MD (2007). The relationship between compensable status and long-term patient outcomes following orthopaedic trauma. Medical Journal of Australia.

[CR4] Harris IA, Murgatroyd DF, Cameron ID, Young JM, Solomon MJ (2009). The effect of compensation on health care utilisation in a trauma cohort. Medical Journal of Australia.

[CR5] Harris IA, Young JM, Rae H, Jalaludin BB, Solomon MJ (2008). Predictors of general health after major trauma. Journal of Trauma.

[CR6] Hickling EJ, Blanchard EB, Buckley TC, Taylor AE (1999). Effects of attribution of responsibility for motor vehicle accidents on severity of PTSD symptoms, ways of coping, and recovery over six months. Journal of Traumatic Stress.

[CR7] Ho R, Davidson G, Van Dyke M, Agar Wilson M (2000). The impact of motor vehicle accidents on the psychological well-being of at-fault drivers and related passengers. Journal of Health Psychology.

[CR8] Hulst, J. E., Akkermans, A. J., & Van Buschbach, S. (2014). Excuses aan verkeersslachtoffers. Een onderzoek naar baten, effectiviteit en methode van het bevorderen door verzekeraars van het aanbieden van excuses aan verkeersslachtoffers [Apologies to transport accident victims. Research on benefits, effectiveness and methods of stimulating the offering of apologies to transport accident victims]. Den Haag: Boom Lemma uitgevers.

[CR9] Littleton SM, Hughes DC, Poustie SJ, Robinson BJ, Neeman T, Smith PN, Cameron ID (2012). The influence of fault on health in the immediate post-crash period following road traffic crashes. Injury.

[CR10] O’Donnell ML, Creamer MC, McFarlane AC, Silove D, Bryant RA (2010). Does access to compensation have an impact on recovery outcomes after injury?. Medical Journal of Australia.

[CR11] Renssen MR (2002). Traumatherapie na verkeersongevallen: Eye movement desensitization and reprocessing (EMDR) bij verkeersslachtoffers [Trauma therapy after traffic accidents: Eye movement desensitization and reprocessing (EMDR) in road casualties.

[CR12] Ruseckaite R, Gabbe B, Vogel AP, Collie A (2011). Health care utilisation following hospitalisation for transport-related injury. Injury.

[CR13] Sullivan MJ, Davidson N, Garfinkel B, Siriapaipant N, Scott W (2009). Perceived injustice is associated with heightened pain behavior and disability in individuals with whiplash injuries. Psychological Injury and Law.

